# Two-Phase Conceptual Framework of Phosphatase Activity and Phosphorus Bioavailability

**DOI:** 10.3389/fpls.2022.935829

**Published:** 2022-07-19

**Authors:** Aamir Manzoor, Michaela A. Dippold, Sebastian Loeppmann, Evgenia Blagodatskaya

**Affiliations:** ^1^Biogeochemistry of Agroecosystems, University of Goettingen, Goettingen, Germany; ^2^Geo-Biosphere Interactions, Department of Geosciences, University of Tuebingen, Tuebingen, Germany; ^3^Institute of Plant Nutrition and Soil Science, Christian–Albrechts University, Kiel, Germany; ^4^Helmholtz Centre for Environmental Research–UFZ, Halle (Saale), Germany

**Keywords:** phosphatase-soil interactions, substrate catalysis, root exudation, mucilage, LMWOAs, phosphatase adsorption

## Abstract

The activity of extracellular phosphatases is a dynamic process controlled by both plant roots and microorganisms, which is responsible for the mineralization of soil phosphorus (P). Plants regulate the availability of soil P through the release of root mucilage and the exudation of low-molecular weight organic acids (LMWOAs). Mucilage increases soil hydraulic conductivity as well as pore connectivity, both of which are associated with increased phosphatase activity. The LMWOAs, in turn, stimulate the mineralization of soil P through their synergistic effects of acidification, chelation, and exchange reactions. This article reviews the catalytic properties of extracellular phosphatases and their interactions with the rhizosphere interfaces. We observed a biphasic effect of root metabolic products on extracellular phosphatases, which notably altered their catalytic mechanism. In accordance with the proposed conceptual framework, soil P is acquired by both plants and microorganisms in a coupled manner that is characterized by the exudation of their metabolic products. Due to inactive or reduced root exudation, plants recycle P through adsorption on the soil matrix, thereby reducing the rhizosphere phosphatase activity. The two-phase conceptual framework might assist in understanding P-acquisition (substrate turnover) and P-restoration (phosphatase adsorption by soil) in various terrestrial ecosystems.

## Introduction

The total soil phosphorus (P) pool (e.g., 224–6,725 kg per ha) consists of organic and inorganic forms of P, of which 80% are immobile and not readily available to plants (Menezes–Blackburn et al., 2013). The organic fraction (P_o_) of soil P consists of dead plant and animal residues, representing 30–65% of the total soil P (Lu et al., 2020). The P_o_ is not bioavailable unless it is mineralized to orthophosphate (${\text{PO}}_{4}^{3-}$ or its protonated forms) (Wang et al., 2021). The remaining 35–70% of soil P pool is inorganic P (P_i_) and is found as insoluble forms of primary (e.g., apatite, smectite, and variscite) and secondary phosphate minerals of calcium (Ca), iron (Fe), and aluminum (Al) that cannot be absorbed by plants until mobilized (Richardson et al., 2009; Pizzeghello, 2011). The availability of P in the rhizosphere is greatly affected by several biophysical processes, including the catalysis of soil P_o_ by extracellular phosphatases, which releases P_i_ (Oehl et al., 2001; Nannipieri et al., 2011). Phosphatase activity can be considered a dynamic process that responds to both root and soil microbial activities, as well as abiotic environmental conditions such as temperature, moisture, and soil adsorption characteristics (Schachtman et al., 1998; Dalling et al., 2016; Tang and Riley, 2021). The root penetration and rhizodeposition processes during nutrient uptake alter both the spatial and temporal distributions of pore spaces in the soil (Crawford et al., 2005; Hill et al., 2015). The altered soil pore space geometry also affects soil porosity and the pathways for transporting nutrients, water, and metabolic products (Hinsinger et al., 2008; Peth et al., 2008; Pettridge and Firestone, 2017). The volumetric water content and structural heterogeneity of the soil influence enzyme activity (Reed et al., 2015; Benard et al., 2019a). As volumetric water content increases in soil, tortuosity and fragmentation of the liquid phase are reduced, facilitating the diffusion of both enzymes and substrates; thus, both molecules can meet, and the substrate is enzymatically catalyzed (Allison et al., 2011; Ali et al., 2015; Ahmed et al., 2018).

On an ecosystem scale, plants acquire soil P through complex interactions with biotic (e.g., soil microorganisms) and abiotic (e.g., soil mineral surfaces) competitors. It has been demonstrated that plant roots and soil microorganisms (e.g., mycorrhizal fungi and their bacterial partners) form a symbiotic relationship that facilitates the hydrolysis of soil P_o_ by soil phosphatases (Haque and Dave, 2005). The rhizosphere's physicochemical conditions are influenced by root exudation of metabolic products [e.g., mucilage and low-molecular weight organic acids (LMWOAs)], affecting the P availability (Araujo et al., 2012). For example, mucilage secretion facilitates root P uptake by modifying soil physical characteristics, such as increasing soil hydraulic conductivity and decreasing P_i_ adsorption to soil surfaces (Zarebanadkouki et al., 2019). Mucilage also affects phosphatase activity by establishing a moist biofilm-like environment (Ahmed et al., 2018; Bilyera et al., 2022). Similarly, LMWOAs have been shown to enhance the desorption of sparingly soluble phosphate monoesters *via* acidification, chelation, and exchange reactions, thus acting synergistically with phosphatases in mineralizing soil P_o_ (Lambers et al., 2015; Koester et al., 2021).

Until now, the microbiology and biochemistry of the root–soil interface have not been sufficiently discussed in relation to root architecture and the physicochemical properties of zones adjacent to the roots. This review aimed to analyze the effects of root exudates and soil abiotic environment on phosphatase dynamics and provide answers to the following research questions (RQs).

RQ_1_: How do plants and microorganisms acquire soil P by releasing their metabolic products, which in addition to their direct role indirectly contribute to the acquisition of soil P by altering soil structure and structure-dependent processes?
RQ_2_: How do soil physicochemical properties influence phosphatase activity (e.g., by adsorption, immobilization, and inhibition), which lead to considerable changes in phosphatase catalytic properties?
RQ_3_: How can phosphatase activity be incorporated into a conceptual framework for quantifying P cycling in soil *via* processes, such as soil P_o_ hydrolysis and phosphatase adsorption to soil matrix?

**Figure 1:**
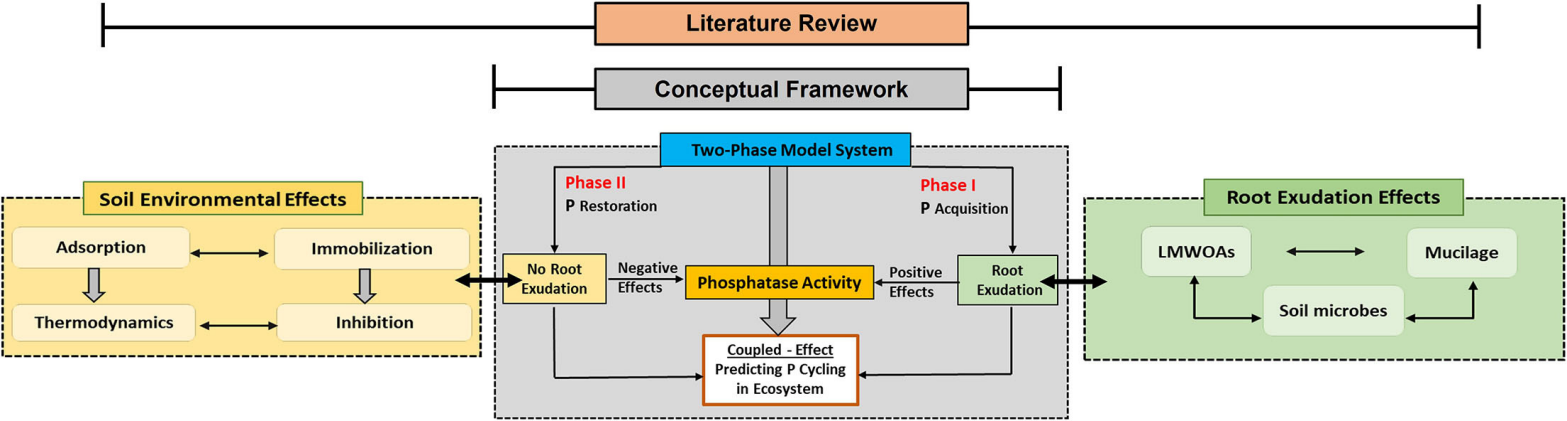
A conceptual two-phase framework that integrates root exudation and soil environment as main drivers of soil phosphatase activity. The dotted lines in each block of main sections indicate their combined effects on phosphatase activity. Based on the conceptual framework block, Phase I shows the soil P_o_ acquisition pathway that is regulated by active root exudation, while Phase II shows soil P_o_ restoration in the absence or with reduced root exudation effects. Both phases are controlled by the P demand of plants with the initiation of P mining processes (green boxes) or by reducing C investment into P mining (yellow boxes).

In order to address these questions, we described the effects of root and microbial exudates on phosphatase activity, examined the interaction of phosphatases with colloidal and mineral soil surfaces, and presented a conceptual two-phase framework that can be used to interpret soil P cycling (Figure 1).

## Effects of Root Exudation on Phosphatase Activity

A plant utilizes 20–40% of its photosynthetically fixed carbon (C) through root exudation to expedite nutrient uptake by supporting the growth of beneficial microorganisms, e.g., symbionts (Badri and Vivanco, 2009). Phloem metabolites (e.g., sugars, amino acids, and LMWOAs) are exuded *via* (i) passive diffusion, (ii) exudation mediated by transporter channels, and (iii) active transport across plasma membranes (Oburger and Jones, 2018). The exuded metabolic products are used by plants to sense nutrient availability relative to their demand at different physiological stages (Canarini et al., 2019). The efflux of exudate into the soil is carried out by plasmodesmata, which connect the cytoplasm of neighboring cells to establish living bridges between them (Ross–Elliott et al., 2017). The physiological mechanisms regulating exudation efflux are strongly affected by external environmental stimuli and exuded metabolic products (Williams and de Vries, 2019; Korenblum et al., 2020). The concentration of primary metabolites present in the root tip serves as a cue for sensing the soil environment and signaling between roots and shoots in order to modify root growth and nutrient allocation (De Schepper et al., 2013; Hu et al., 2018). The soil microorganisms affect the rate of exudation at the root tips through their consumption and enzymatic transformation of released metabolites (Sasse et al., 2019).

### Low Molecular Weight Organic Acids

The root-exuded LMWOAs contain one to three carboxylic groups; among them, malic and citric acids (>2 mmol kg^−1^ soil) are the most abundant and prevalent organic acids associated with P mobilization (Denton et al., 2007; Aziz et al., 2011; Hunter et al., 2014). A typical concentration of LMWOAs in soil solution ranges between 0–50 μmol for dicarboxylic acids and 0–1 mmol for monocarboxylic acids (Strobel, 2001; Nwoke et al., 2008). Under abiotic stress, root tips of plants regulate malate anion efflux channels (ALMT) that initiate malate exudation in response to P deficiency (Ramesh et al., 2015; Gilliham and Tyerman, 2016; Mora–Macías et al., 2017). A high exudation of citric acid can lower soil pH and enhance P uptake by white lupin (Weisskopf et al., 2006). Citric acid, as a tricarboxylic acid, is more effective than dicarboxylic and monocarboxylic acids at mobilizing soil P (Khademi et al., 2009; Gang et al., 2012). This is because tricarboxylic acids are more capable of (i) forming stable chelation complexes with Ca in alkaline soils, thereby preventing precipitation of P (Kirk et al., 1999), (ii) complexing Al to reduce precipitation of Al–P hydroxy-phosphates, thus promoting weathering of P-bearing rocks (Pearse et al., 2007), (iii) remobilizing adsorbed P from soil surfaces *via* ion exchange, and (iv) preventing adsorption of P by soil matrix (Oburger et al., 2011). The majority of LMWOAs (57%) is consumed by soil microorganisms as part of their metabolic processes (e.g., respiration), while the rest is absorbed by soil colloids (Andrade et al., 2013).

Plant roots and soil microorganisms actively release phosphatases and LMWOAs as a result of ATP consumption for the acquisition of P in the P-deficient rhizospheres (Kelleher et al., 2004; Nannipieri et al., 2011; Ajmera et al., 2019). LMWOA anions either occupy sorption sites on the soil mineral surfaces to replace P_i_ or contribute to the hydrolysis of adsorbed P_o_ (Lambers et al., 2015; Wang Y. et al., 2017). Their mutual relationship in mobilizing and mineralizing soil P_o_ is shown to be synergistic in the conceptual model of Clarholm et al. (2015). The bioavailability of P depends on the interaction of pH, phosphatase activity, and the concentration of LMWOAs in the soil solution (Adeleke et al., 2012). For example, a significant positive correlation was found between pH and phosphatase activity in the rhizosphere of drought-tolerant and non-drought-tolerant corn varieties under water stress (Song et al., 2012). The authors observed that osmotic stress increased the concentration of LMWOAs exuded by roots, resulting in increased phosphatase activity during elongation, tasseling, and filling stages, whereas drought-tolerant corn varieties showed higher phosphatase activity. Similarly, glucose, glutamate, and citrate were found to significantly increase phosphatase activity in both clayey and sandy soils at pH 6.9 and 5.1 (Renella et al., 2007). A significant increase in phosphatase activity was observed in the rhizospheres of *Crotalaria juncea* and *Tithonia diversifolia* at a distance of 0–1 mm from the root surface (George et al., 2002). The above examples illustrate a close relationship between LMWOA exudation and phosphatase activity in promoting phosphatase-mediated P_o_ mineralization by multiple mechanisms.

### Root Mucilage

Mucilage secreted by the plant roots is composed of polysaccharides or long-chain sugar molecules and proteins that form a gelatinous substance that adheres to the root cap (Bais et al., 2008). As a result of mucilage secretion, a biopolymer layer forms in the soil around the root tips, known as rhizosheath structures (Delhaize et al., 2012). Rhizosheath structures are correlated to the length and density of the root hairs and modify the rhizosphere transport processes (George et al., 2014; Pausch et al., 2016). The gelatinous nature of mucilage facilitates the uptake of water and nutrients by increasing the hydraulic conductivity of soil particles and binding them together and with the roots (Kroener et al., 2014; Carminati et al., 2016). Mucilage, with its water absorption properties, affects soil hydraulic properties due to the presence of surfactants (Zickenrott et al., 2016). Mucilage can absorb water up to 1,000 times its dry weight to maintain a moist rhizosphere where bulk soil dries out more quickly (McCully and Boyer, 1997). The decrease in volumetric water content of the soil reduces surface tension and increases mucilage viscosity, which improves its ability to sustain liquid bridges across soil particles (Carminati et al., 2017; Benard et al., 2019b).

Phosphatase activity is closely related to root mucilage and its ability to increase hydraulic conductivity in soil pores (Jones et al., 2009). Due to mucilage secretion, rhizosphere volumetric water content is higher than bulk soil. These effects enhance enzyme diffusion into the root zone, creating a hotspot of enzymatic activity (Carminati et al., 2010; Manzoni et al., 2014). For example, a reduction in volumetric water content in the rhizosphere of barley caused a 97% decline in phosphatase activity, demonstrating a strong and reversible impact on soil phosphatase activity (Holz et al., 2019). In another study, a low concentration of mucilage secreted by maize roots (40 μg C g^−1^ soil is equivalent to 10% of microbial biomass C) significantly increased the soil phosphatase activity under drought (30% of soil water holding capacity) (Ahmed et al., 2018). In the presence of a high mucilage concentration (200 μg C g^−1^ soil is equivalent to 50% of microbial biomass C), the drought effect was overcompensated, leading to a one-third increase in phosphatase activity. Plants increase phosphatase activity directly by producing and exuding phosphatases through roots and indirectly by providing labile C, as found in root mucilage, to promote microbial activity (Rejsek et al., 2012; Spohn and Kuzyakov, 2013). The increase in viscosity of mucilage under drought conditions causes it to transform into a hydrophobic inter-particulate glue that strongly inhibits the diffusion of substrates toward phosphatases, resulting in decreased availability of P_i_ to plants (Hunter et al., 2014; Brax et al., 2020). Microorganisms in thin water films around soil particles may suffer dehydration and go dormant (as cysts or spores) or even die as a result of denaturation of their cellular components under osmotic stress (Schimel and Balser, 2007; Williams and Rice, 2007; Loeppmann et al., 2018).

### Substrate–Product–Enzyme Interactions and Their Kinetic Description

The economic model of extracellular enzyme production treats soil microbial communities as economic units during their resource allocation to produce C-, N-, and P-releasing enzymes (Sinsabaugh and Moorhead, 1994). In spite of contrasting soil properties and nutrient stocks, the C- and P-cycling enzyme network enhanced nutrient acquisition to maintain microbial growth, which indicates a similar trade-off between C- and P-cycling enzymes (Loeppmann et al., 2020). Extracellular phosphomonoesterase activity regulates the P forms in soil and depends on the enzyme production itself, such as the release of the enzyme by plants and microbes into the soil solution, or the availability of labile P substrate (Turner and Haygarth, 2005; Burns et al., 2013). Total composition and quality of soil P_o_ influence enzyme- and substrate-dependent catalysis of soil P_o_ compounds (Quiquampoix and Mousain, 2005; Noll et al., 2019). Both acid and alkaline phosphomonoesterases hydrolyze phosphomonoesters, e.g., inositol phosphates and phytins, which constitute between 20 and 50% of the total soil P (Dalal, 1977; Turner, 2008). Compared with phosphomonoesterases, phosphodiesterases exhibit relatively low activity (e.g., in acidic soils) as a result of the resiliency of phosphodiesters to degradation or sorption, both due to their protected phosphate ester groups (Jarosch et al., 2019). The phosphodiesters found in fresh detritus of plant or microbial origin have a low persistence in the soil and rarely exceed 1% of the total soil P (Paul and Clark, 1989). The activities of phosphomonoesterases and phosphodiesterases cannot be separated completely. The phosphodiesterase produces highly labile substrates for the phosphomonoesterase, which, in turn, releases orthophosphate, which is a potential inhibitor for both enzyme groups (Leake and Miles, 1996).

In nature, enzyme-driven substrate catalysis occurs spontaneously and is primarily regulated by the diffusion of substrate monomers toward enzymes present in solution or adsorbed at the soil surface (Datta et al., 2017). The breakdown of soil P_o_ by phosphatases involves the production of metaphosphate as an intermediate product, which is then converted into orthophosphate in the presence of water (Lassila et al., 2011). As a result of the rapid protonation of phosphoryl groups, phosphate-monoester anions have increased electrophilicity, which allows them to react more quickly with substrates in contrast to phosphate-diester anions (Hengge, 2005). Experimental evidence concerning the co-occurrence of phosphatase activity and the P depletion zone has been obtained up to a distance of 2–4 mm from the root surface (Nuruzzaman et al., 2006; Hummel et al., 2021). Phosphatase activity near the root surface reduces the concentration of P_o_ substrate compared with the surrounding soil (Burns et al., 2013). As a result, a concentration gradient drives substrate diffusion from solution, while catalysis depends largely on phosphatases present in a free state or adsorbed on the soil surfaces. The rate of product formation and/or the rate of substrate catalysis (*v*) can be determined by the Michaelis–Menten Equation (1).


(1) $$\begin{array}{l} v=\;\frac{V_{\max}\;\times \;S}{K_{m}\;+S}, \end{array}$$

where *V*_*max*_ is the maximum reaction rate of product formation and *K*_*m*_ is the Michaelis constant, which indicates the affinity of enzymes to specific substrates (e.g., *K*_*m*_ is inversely proportional to affinity) and is defined as substrate concentration (*S*) at half of *V*_*max*_.

The kinetic parameters of the substrate-dependent Michaelis–Menten model are merely the weighted means of the characteristics of the enzymatic activity catalyzed by many diverse enzyme systems in soil; hence, they are often defined as apparent *V*_*max*_ and *K*_*m*_. However, the Michaelis–Menten approach in environmental modeling remains important for understanding the effects of plant–microbial interactions and soil physicochemical conditions on average substrate turnover rates (Eberwein et al., 2017).

### Plant Photosynthesis and Microbial P Acquisition: A Coupled Relationship

**Figure 2:**
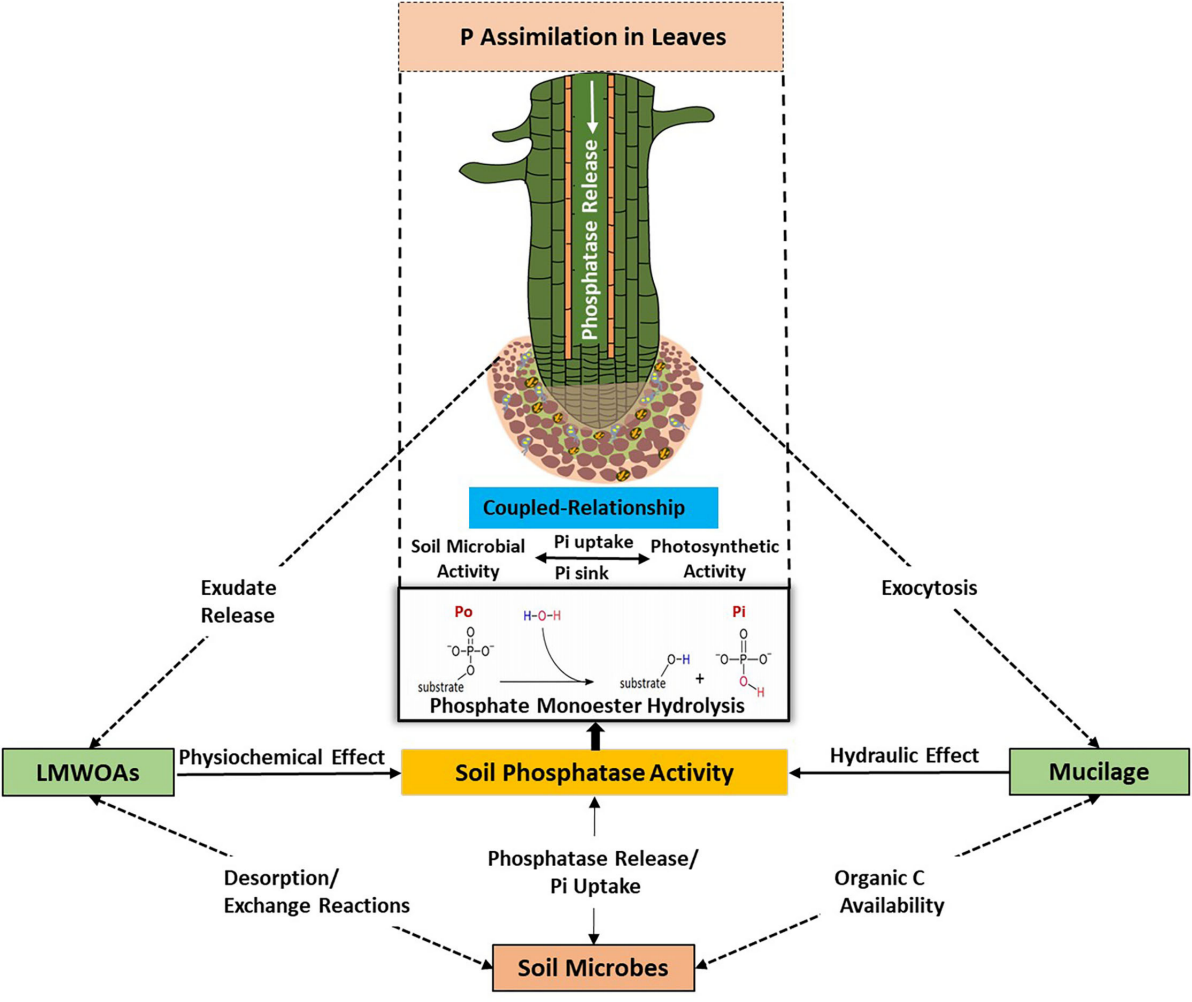
A scheme visualizing the coupled relationship between plants and soil microbes for the acquisition of available soil P resources and/or P mineralization induced *via* phosphatase activity. Root exudation of mucilage and LMWOAs exert favorable physicochemical and hydraulic changes in the rhizosphere for P accessibility. Soil microbes ensure a mutual relationship with plant roots by the production of phosphatases and release of LMWOAs, which favor P supply from soil P reservoir.

Root secretion of mucilage and exudation of LMWOAs is carried out by passive and active mechanisms, respectively (Jones et al., 2009). Together with dying root cells, the released C sources serve as microbial substrates that promote microbial activity and growth (Loeppmann et al., 2016a,b). A coupled relationship has been observed between photosynthetic activity and root exudation, which is mediated by soil microorganisms through consumption and biotransformation of released metabolites (Doan et al., 2017; Vidal et al., 2018). For example, the photosynthetic P-use efficiency was found to be exceedingly high for Proteaceae and non-Proteaceae species with different leaf traits (Pereira et al., 2019). On nutrient-poor sites, P was used much more efficient for photosynthesis, indicating a species-independent increase in P-use efficiency in both Proteaceae and non-Proteaceae with decreasing soil P availability (Pereira et al., 2019). The competitive saturation of sorption sites by organic C anions caused by elevated organic C content of soil led to an increase in the lability, solubility, and transport of P_i_ in soil (Reddy et al., 1980; Ohno and Crannell, 1996; Brucker et al., 2020). The low availability of dissolved P_i_ in soil solution (<0.01–1 mg L^−1^) results in starvation conditions for soil microbes, thus they have to use multiple strategies to acquire P, such as improving P assimilation, optimizing intracellular P metabolism, and mobilizing extracellular P (Steinweg et al., 2013; Grafe et al., 2018; Pistocchi et al., 2020). We propose a coupled relationship that entails the provision of labile C to soil microorganisms through root exudates (e.g., mucilage, LMWOAs) and the microbial response to access soil P through phosphatase activity. This coupled relationship (Figure 2) controls both the uptake of P_i_ by plant roots and soil microorganisms, and hence phosphatase activity, which in turn is determined by phosphatase production and dissolved P_i_ in soil solution.

## Effects of Abiotic Soil Environment on Phosphatase Activity

**Figure 3:**
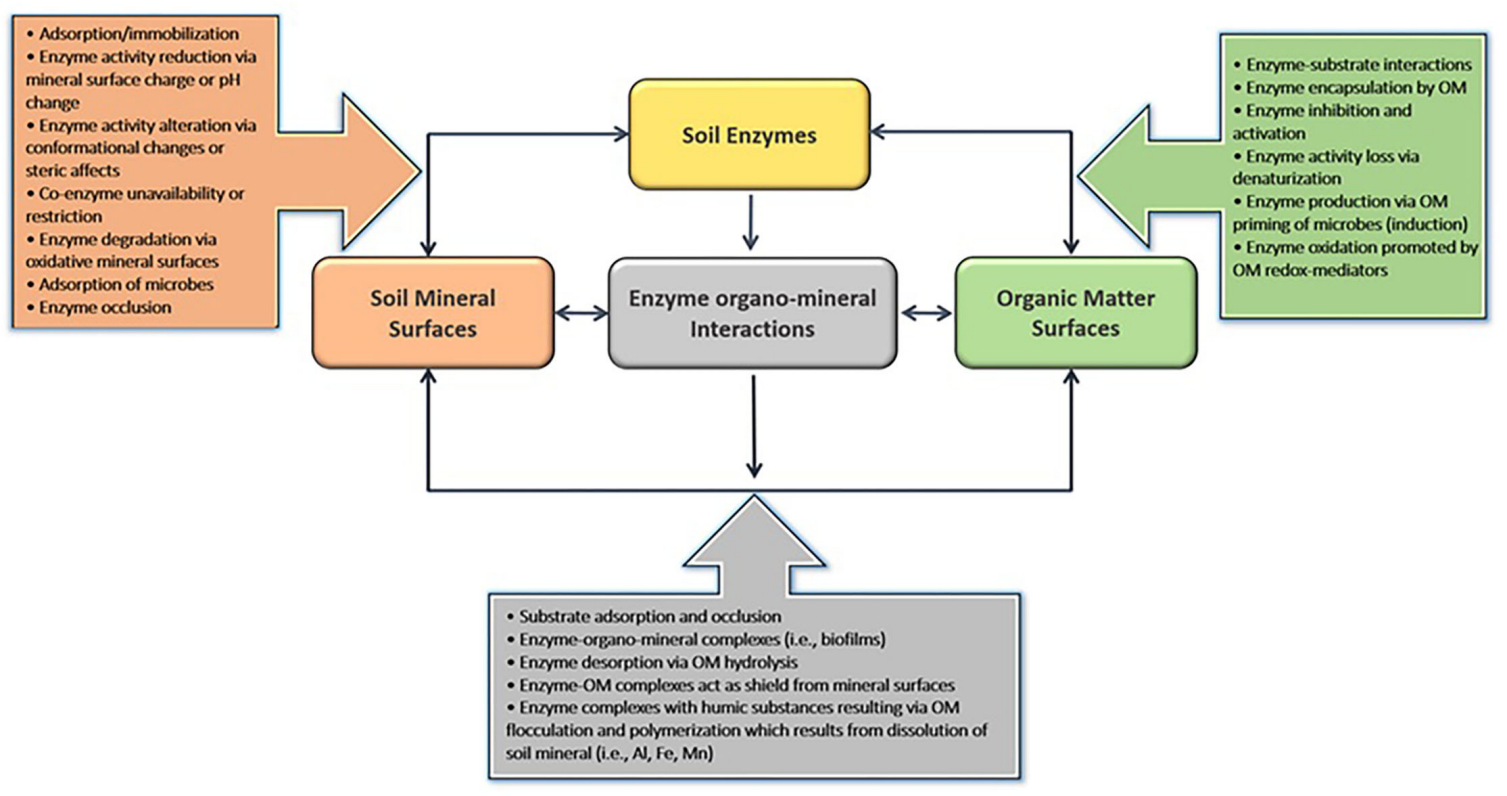
A summary of the interactions between soil colloidal and organic matter surfaces and extracellular enzymes and their effect on enzyme–substrate complex formation.

The abiotic soil environment strongly regulates substrate degradation rates since it influences the nutrient-foraging strategies of soil microorganisms (Schimel and Weintraub, 2003; Gianfreda, 2015). After being released from plant roots as exudates or microbial secretions, extracellular phosphatases form many types of associations (Figure 3), including (i) enzyme–substrate complexes, (ii) adsorption to clay minerals, (iii) complexing with soil organic matter surfaces through entrapment, absorption, or co-polymerization, and/or (iv) present in freely diffusible forms in soil solution (Wallenstein and Burns, 2011).

### Phosphatase Adsorption on Colloidal and Mineral Soil Surfaces

In soil, extracellular phosphatases are present in solution or they bind reversibly to soil colloidal and mineral surfaces, which protect them against microbial decomposition and exposure to environmental stresses (Burns, 1982; Kedi et al., 2013; George et al., 2014). Several mechanisms can account for the strong adsorption affinity of phosphatases for soil colloidal components (<2 μm), including electrostatic Van der Waals forces, Lewis's acid–base effects, hydrophobic interactions, and conformational entropy (Ikeda and Kuroda, 2011). In the rhizosphere, organic and inorganic ligands (e.g., LMWOAs and phosphate anions) contribute to phosphatase adsorption through ion exchange, covalent complexation, and hydrogen bonding (Nannipieri et al., 1988). The abundance of silica (SiO_2_) in soil facilitates the physicochemical interaction of phosphatases with soil surfaces through the negative charge of silanol groups (Si–OH) and siloxane bridges (–Si–O–Si–) (Zhuravlev, 2000). Typically, free or colloid-associated phosphatases are transported to soil surfaces by convection (laminar or turbulent flows); however, their final adsorption to soil surfaces is carried out by diffusion (Datta et al., 2017; Guber et al., 2022). Due to electrostatic attraction, oppositely charged phosphatases adopt a spatial orientation toward soil surfaces during adsorption (Margalef et al., 2017). The phosphatase adsorption by colloidal and mineral surfaces of soil can be explained by Langmuir's Equation (2).


(2) $$\begin{array}{l} X=\frac{X_{m}\;\times \;C}{K\;+\;C}, \end{array}$$

where *X*_*m*_ is the maximum adsorption capacity of enzymes, *C* is the equilibrium concentration of enzymes in solution, and *K* is the binding energy of enzyme molecules (e.g., low *K* values indicate high affinity of enzymes for colloids and minerals) (Huang et al., 2003). Langmuir's equation assumes uniform surface area and is commonly used to estimate phosphate adsorption, but it can underestimate the amount of adsorption since it neglects multiple possible sorption pathways (Hussain et al., 2012; Kruse et al., 2015).

In acidic soil environments, phosphatases adsorb to finer colloidal particles (e.g., goethite and montmorillonite), which can be exacerbated by the presence of iron oxides with large surface areas and high anion exchange capacity (Huang et al., 2005; Zhao et al., 2012). Organic components (e.g., humic acids) facilitate phosphatase adsorption by trapping them within their macromolecular networks (Kelleher et al., 2004). Adsorption of phosphatase by LMWOAs and inorganic anions decreased in the following order: phosphate > tartrate > oxalate > acetate (Huang et al., 2003). Nevertheless, addition of acetate (0–10 mmol) increased phosphatase adsorption onto goethite and kaolinite surfaces by protonating the surfaces of the acetate adsorbates, thereby creating more adsorption sites. As anion adsorption density increased, the promoter effect of acetate diminished at concentrations above 10 mmol as a result of steric and competitive interactions (Kafkafi et al., 1988; Geelhoed et al., 1998). By contrast, higher concentrations of oxalate (0–50 mmol) reduced phosphatase adsorption on goethite surfaces (Zhao et al., 2012). Oxalate affects the adsorption properties of clay minerals by forming mono- (pH 3.5) and di-coordinate (pH 4.5 and 5.5) complexes that reduce phosphatase adsorption through conformational changes (Bhatti et al., 1998). Phosphatases maintain a neutral charge at their isoelectric point (pH~5), which is necessary to preserve their natural molecular configuration (Huang et al., 2003). A reduction in adsorption at the isoelectric point would facilitate the achievement of maximum specific activity by reducing the congestion among phosphatase molecules. When pH decreases from 5, an electrostatic repulsion develops between phosphatases and clay minerals (both being positively charged), resulting in reduced adsorption (Leprince and Quiquampoix, 1996). In addition, the negative charge on the surface of phosphatases and clay minerals above the isoelectric point inhibits adsorption, thereby allowing the phosphatases to diffuse into the water-filled pores of the soil. We therefore speculate that phosphatase activity and adsorption may be affected by the competitive adsorption of enzymes and organic/inorganic anions. Both mechanisms (phosphatase–P_o_ substrate and phosphatase–soil interactions) are controlled by changes in enzyme configuration/coverage on soil colloids and organic anion-induced dissolution of soil colloids and minerals.

### Phosphatase Immobilization and Inhibition Kinetics

Adsorption of enzymes generally prevents their degradation since immobilized enzymes are less susceptible to environmental stresses, because their 3D structure is stabilized through the surface–surface interactions, and stressors are prevented from accessing them (Joner and Johansen, 2000). Due to immobilization, phosphatase activity is largely controlled by pH fluctuations, temperature variations, structural orientation effects, and steric hindrances caused by organic or inorganic ligands (Demanèche et al., 2009; Kedi et al., 2013). For example, after immobilization by minerals and sediments, alkaline phosphatases showed a 5% decline in activity (Zhu et al., 2016). However, after immobilization by soil inorganic components, acid phosphatases showed distinct patterns of activity in the following order: allophane > kaolin > Fe oxide > montmorillonite > Al oxide = Mn oxide (Shindo et al., 2002). Acid phosphatases immobilized on Al, Fe, Mn oxides, or montmorillonite retained 13–23% of their activity, whereas acid phosphatases immobilized on kaolin and allophane retained 57 and 77% of their activity, respectively. As a result of adsorption, acid phosphatases showed a decrease in *V*_*max*_, an increase in *K*_*m*_, and a decrease in *V*_*max*_/*K*_*m*_ ratio (Shindo et al., 2002). The specific activity and adsorption strength of acid phosphatases increased with the increasing concentration of organic anions (e.g., oxalate) (Zhao et al., 2012). The general consensus is that organic ligands increase phosphatase activity (a stimulatory effect) compared with inorganic ligands (a competitive effect) (Pascual et al., 2002).

The inhibition of phosphatases by immobilization occurs through interactions with colloidal and mineral soil surfaces *via* competitive inhibition, non-competitive inhibition, or mixed mechanisms (Deng and Tabatabai, 1995). Phosphatase inhibition causes the formation of enzyme–substrate, enzyme–inhibitor, or enzyme–substrate–inhibitor complexes (Quiquampoix et al., 2002; Tietjen and Wetzel, 2003). The presence of certain inorganic anions (e.g., phosphate) and heavy metals (e.g., arsenate) may inhibit phosphatases, leading to a reduction in their affinity for substrates (Tian et al., 2018). As phosphatases are synthesized by plants under conditions of P_i_ limitation, their activity is referred to as P_i_-repressible activity (Rombola et al., 2014; Čapek et al., 2021). Phosphatase activity is inhibited by dissolved P_i_ concentrations in solution, suggesting a close relationship between the use of dissolved P_i_ in the soil and the mobilization of plant's internal P reserves (Gianfreda and Ruggiero, 2006; Maseko and Dakora, 2013). A direct, positive relationship exists between phosphatase activity and product concentration when soil biota gains optimal access to dissolved P_i_ under conditions of high P demand (Treseder and Vitousek, 2001). The inverse relationship can be observed when high levels of phosphatases are present without significant formation of products (Allison S. D. et al., 2007; Allison V. J. et al., 2007). In addition, the negative correlation is also evident when phosphatases are adsorbing to colloidal and mineral soil surfaces, e.g., lowering their catalytic efficiency (Weintraub and Schimel, 2005).

Phosphatase immobilization on montmorillonite or goethite surfaces results in a reduced inhibitor affinity by reducing the accessibility of inhibitors to the catalytic sites (Bhattacharyya et al., 2008; Wang Z. Q. et al., 2017). For example, the competitive inhibition represents the direct competition between an inhibitor and substrate at the enzyme's active site, resulting in a decrease in *K*_*m*_ but an increase in *V*_*max*_, indicating that the enzyme's active sites are temporarily bound to the organic matter surfaces (Zimmerman and Ahn, 2010). The ability to characterize the competitive inhibition of phosphatases can be useful in predicting inhibitor toxicity to soil phosphatase activity (Wang et al., 2018). As a result of high inhibitor concentrations, *K*_*m*_ increases, resulting in a more difficult-to-break ESI complex, which reduces *V*_*max*_ (Dick and Tabatabai, 1987; Cornish–Bowden, 2015; Wang Z. Q. et al., 2017). Phosphatases immobilized on mineral soil surfaces exhibit a complete inhibitory effect, whereas free phosphatases in solution show a mixed, linear inhibitory effect (Tian et al., 2018). The reason for this is that inhibitors (e.g., phosphate anions, arsenate) cannot fully compete with the substrate on the active sites of immobilized phosphatases due to their adsorption by mineral soil surfaces. Conversely, inhibitors are better able to compete with the substrate at the active sites of free phosphatases when there are few adsorption sites supplied by the enzyme–substrate complex. A non-competitive inhibition mechanism is characterized by inhibitors binding to the enzyme–substrate complex or enzyme itself, but not the active site, causing a reduction in *V*_*max*_ and no change in *K*_*m*_ (Ahn et al., 2006). In acidic soils, non-competitive inhibition reduces phosphatase activity to a significant extent, but this can be slowly recovered with an increase in organic matter content and cation exchange capacity (Wang et al., 2018). A decline in phosphatase activity in alkaline soils can be attributed to competitive inhibition and non-competitive inhibition effects, with an increase in *K*_*m*_ resulting from increasing inhibitor concentration.

## Conceptual Two-Phase Framework

In a heterogeneous soil environment, diffusion and mass flow-based transport processes co-regulate P_o_ mineralization through extracellular phosphatases. During water flow toward the plant's xylem, mass flow facilitates substrate transport to phosphatases close to the roots, while diffusion governs P_o_ hydrolysis and is dominated by readily available dissolved P_i_ in soil. The fast decomposition of the substrate (P_o_) and the uptake of released products (P_i_) in the immediate vicinity of roots enhance the diffusion of P toward root surfaces. Due to the strong adsorption of P_i_ ions on the soil surfaces, diffusion alone is not sufficient to meet the plant's P requirements. This results in a non-equilibrium shift between high P_i_ uptake and low replenishment from the soil, resulting in P depletion zones that extend from the root surface toward the bulk soil. The production and subsequent activity of extracellular phosphatases are strongly related to the depletion of P_i_ from the soil solution, suggesting that plants meet most of their P needs by mineralizing soil P_o_. In addition, metabolic products that are excreted by the roots (e.g., mucilage and LMWOAs) play an important role in both modifying the soil physicochemical conditions to increase P accessibility and indirectly enhancing extracellular phosphatase activity. In our conceptual two-phase framework, we explain how root-exuded metabolic products, such as mucilage and LMWOAs, regulate phosphatase activity by altering soil physicochemical and hydraulic conditions. It is hypothesized that exudate-imposed conditions can affect soil P bioavailability in response to the P demand of soil biota. An opposite scenario occurs when the active P demand has been met, resulting in reduced exudation of bioactive compounds, which conserves available P resources and phosphatases by adsorption to the soil matrix.

### Mechanistic Interpretation

**Figure 4:**
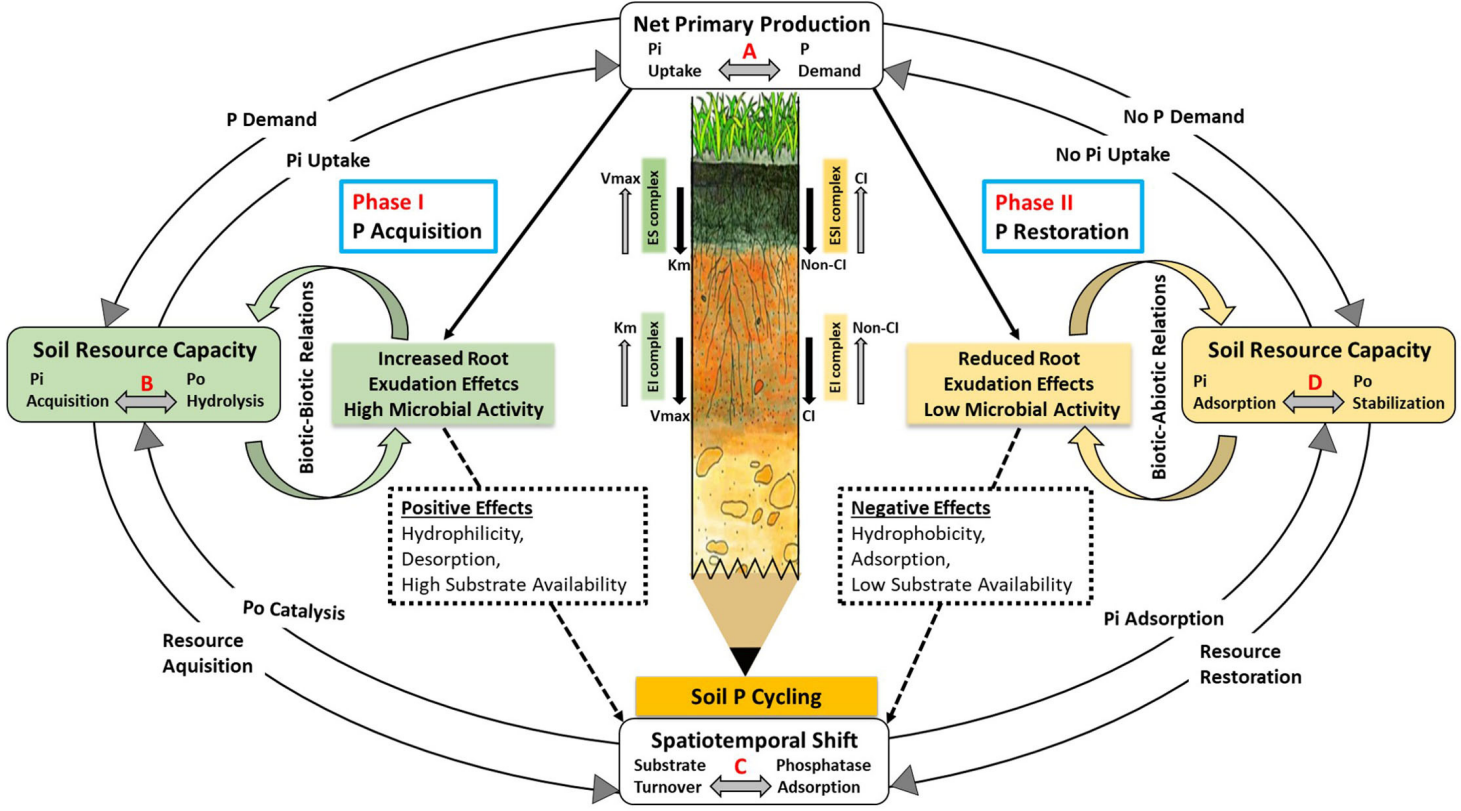
A conceptual two-phase framework for predicting P bioavailability in ecosystems based on phosphatase activity and soil phosphatase interactions. In “Phase I”, plants acquire soil P via phosphatase activity, which is controlled by a coupled relationship (biotic relations) with soil microorganisms in the presence of exuded metabolic products such as mucilage and LMWOAs. The “phase II” is characterized by the conservation of soil P resources under the dominance of biotic and abiotic interaction among microbes, phosphatases, and the different P forms with the soil mineral surfaces in the absence of root exudation. During phase II, extracellular phosphatases are immobilized and adsorbed onto colloidal and mineral soil surfaces, which inhibits their activities through modifications in hydraulic and physicochemical properties. CI, competitive inhibition; non-CI, non–competitive inhibition; ES, enzyme–substrate; EI, enzyme–inhibitor; ESI, enzyme–substrate–inhibitor.

The framework aimed to address the challenges involved in developing efficient, reliable, and useful P models for ecosystem studies. To provide a mechanistic explanation, a two-phase dynamic system (Figure 4) is constructed to describe the acquisition of soil P by plants *via* root exudation and the conservation of soil P *via* adsorption. Both phases are influenced by the presence or absence of stimulating root exudates.

The proposed two-phase model system is composed of several interrelated processes marked with letters A, B, C, and D. The processes designated with the letter “A” are connected with the P demand of plants, which in turn facilitates root uptake of dissolved P_i_ through diffusion, which is relatively low (0.1–5 × 10^−13^ m^2^ s^−1^) as compared to other nutrients (Bhadoria et al., 1991). Due to a continuous uptake of P_i_ by plant root systems, a rapid equilibrium between dissolved P_i_ and labile soil P_i_ fractions is disturbed, leading to a faster release of P_i_ into solution (Tinker and Nye, 2000; Mengel et al., 2001). Those processes with the letter “B” involve an increase in the P requirement of plants due to a low level of dissolved P_i_, leading to physiological changes in plants. Consequently, plants exude metabolic products (e.g., LMWOAs and phosphatases), which facilitate the mobilization of sparingly soluble soil P forms and/or the mineralization of soil P_o_ (Chen et al., 2002; Merlin et al., 2016). The hydrolysis of soil P_o_ is inhibited when dissolved P_i_ is no longer the limiting factor for plant uptake, suggesting a negative relationship between P_i_ availability and phosphatase activity (Moscatelli et al., 2005; Marklein and Houlton, 2012). The letter “C” represents catalytic processes involving P_o_ mineralization and/or phosphatase soil adsorption in the absence of root-exuded metabolic products. The coregulation of labile P_i_ uptake and P_o_ mineralization is contingent on the plant's P demand, which then drives the system either toward P-mineralization or microbial P-immobilization (Zhang et al., 2018; McConnell et al., 2020). The letter “D” denotes processes that are executed under P restricted conditions that may result in P immobilization (e.g., increasing P storage in microbial cells), which will eventually result in the transition from P acquiring systems to an efficient P recycling system (Lang et al., 2017; Manzoni et al., 2021). The decrease in P demand for immobilization by plants and soil microbes also results in a decrease in the exudation of their metabolic products, including the production and exudation of extracellular phosphatases (Rejsek et al., 2012). Following the cessation of exudation of metabolic products (e.g., mucilage, LMWOAs) from plants' roots, the rhizosphere conditions modify to trigger P immobilization pathways involving adsorption of P_i_, P_o_, and phosphatases to soil surfaces.

#### Phase I: P Acquisition by Increased Root Exudation Effects

The secretion of mucilage at the root tips has multiple functions, including (i) mobilizing P_i_ from mineral sorption sites, (ii) transporting mobilized P_i_ to the roots, and (iii) facilitating the mineralization of Soil P_o_ by extracellular phosphatases. Mucilage stimulates the growth and activity of soil microorganisms by providing them with labile C, thereby indirectly increasing phosphatase activity. The texture of mucilage gel promotes the retention of more water during drought events, so mucilage increases soil hydraulic conductivity. When the soil dries out, water diffuses from the gel and forms liquid bridges with soil surfaces that encapsulate root tips. These liquid bridges act as small water channels connecting the soil particles with each other and with the surface of the roots to facilitate the transport and uptake of P_i_. In situations of high P demand, mucilage reduces the diffusion barrier of phosphate monoesters and allows them to reach phosphatases near the roots. The increased secretion of mucilage reduces its viscosity, which increases the surface tension of the solution directly attached to the soil pore surfaces. Ultimately, this increases the hydrophilicity of phosphatase catalytic active sites required for P_o_ catalysis and enhances phosphate desorption from soil surfaces. Mucilage gels are capable of retaining water, creating a hydrodynamic barrier that can restrict the diffusion of phosphatases, thereby preventing their adsorption at soil surfaces. The catalytic efficiency of phosphatases increases with desorption resulting in the formation of an ES complex as indicated by a high *V*_*max*_ and a low *K*_*m*_. In summary, mucilage secretion can either increase or decrease phosphatase activity by increasing soil hydraulic conductivity and desorbing phosphate anions. Similarly, root-exuded LMWOAs alter soil physicochemical properties in two ways: they (i) increase P bioavailability through the solubilization of crystalline and/or amorphous P-containing minerals and (ii) function synergistically with phosphatases through anion-exchange reactions. This synergistic relationship results in (i) the blocking of phosphatase adsorption sites at the soil surface, which helps to reduce (re-) adsorption and (ii) the introduction of pH changes to the isoelectric point of phosphatases, which increases their activity. A net neutral charge on phosphatases is ensured by the isoelectric point, which maintains their natural molecular configuration during the formation of ES complexes. A maximum level of phosphatase activity is observed between pH values of 3 and 5, which subsequently increases due to root exudation of LMWOAs. At pH < 3, exuded LMWOAs represent an indirect source of competitive inhibition (e.g., desorption of P_i_ and inhibitor ions from soil surfaces), thereby decreasing the substrate accessibility to phosphatases. Similarly, phosphatase soil adsorption reaches its maximum level below the isoelectric point and decreases at a low pH. Both phosphatases and adsorbing soil surfaces are positively charged at low pH, resulting in a strong electrostatic repulsion, which may account for phosphatase desorption in acidic soils.

#### Bridging Phase: A Spatiotemporal Shift in Root Activity

Phase I and its underlying processes are continued until P_i_ availability is no longer a limiting factor for plant uptake. The acquisition of dissolved P_i_ by plant roots depends on many factors, including (i) the P demand of plants at each stage of their physiological development, (ii) the soil physicochemical properties, and (iii) the environmental factors associated with P accessibility to plants (e.g., water availability). Our hypothesis states that the P demand of plants is not limited by excessive dissolved P_i_, but it is shifted toward alternative locations during root development in the soil. Root exudation activity may improve effective utilization of dissolved P_i_ through reallocation of soil P resources during root growth. The term “effective utilization” refers to acquiring soil P_i_ in a way that is advantageous over the addition of exuded metabolites. Plant roots secrete mucilage and exude LMWOAs, which contribute to effective soil P acquisition. These processes may also have residual effects due to spatiotemporal shifts in root activity while the roots are still growing in the soil. In our opinion, either the plants' P requirement has been met at the “depleted hotspots” or there is a decline in root exudation activity as a result of environmental factors (e.g., water scarcity). In phase II of the current framework, we refer to these residual rhizosphere effects as “depleted root activity” effects that contribute to P restoration in the soil matrix.

#### Phase II: P Restoration by Reduced Root Exudation Effects

The spatiotemporal shifts encountered during root growth result in reduced P demand of plants at individual sites, and as a consequence, reduced root exudation activity, thus leading to the “depleted root activity effects” effects. These effects increase during drought, disrupting the coupled relationship between plant roots and soil microorganisms that was established during phase I. When root exudation is reduced, microorganisms struggle to mobilize sufficient P, resulting in a joint C and P deficiency, which results in a reduced level of activity and growth. The resulting effect is the interaction between soil microbes and the mineral surfaces of the soil to maintain the basic requirement of microbial P acquisition. The transition to microbial dormancy when nutrients are scarce starts phase II of our conceptual framework leading to soil P restoration. Drought accelerates P restoration through the adsorption of extracellular phosphatases and P_i_ anions, as well as labile soil P_o_ as high-quality substrates for future reserves when soil location shifts toward a phase I condition. A decrease in mucilage secretion reduces hydraulic conductivity and soil pore connectivity, which in turn has a negative effect on the transport and uptake of dissolved P_i_, as well as its desorption from the soil surfaces. The decrease in hydraulic conductivity as a result of low soil volumetric water content restricts the transport of phosphatases and substrate to thin water films covering the soil pore surfaces. In the absence of fresh mucilage secretion by plant roots, the concentration of mucilage in the rhizosphere increases due to drying, which increases viscosity and decreases surface tension within the biogel. As a result of the lower surface tension, the hydrodynamic barrier between phosphatases and adsorbing soil surfaces is reduced, and phosphatases adsorb to soil surfaces at different concentration gradients. During Phase I, this concentration gradient exists between adsorbed phosphatases and product concentrations (dissolved Pi) in the soil solution and serves as a source of competitive inhibition for phosphatases. During Phase II, this gradient exists between adsorbed phosphatases and their concentration near the soil surfaces. Moreover, a reduction in LMWOAs exudation by plant roots may result in an increase in phosphatase adsorption, since LMWOAs desorption frees up phosphatase adsorption sites. In the event of a pH rise above the isoelectric point (>5), a negative charge is generated, causing electrostatic repulsion between phosphatases and soil colloids. Consequently, the presence of high alkalinity also results in an increase in inhibitor concentration in solution, which leads to a competitive inhibition that reduces substrate accessibility to phosphatases, leading to the formation of the enzyme–inhibitor or enzyme–substrate–inhibitor complexes.

The P restoration phase is in effect until the “depleted root activity” effects are mitigated by increasing the P demand of plants, thereby transitioning from phase II to phase I. The dynamic spatiotemporal shifts between both phases are considered essential to our cyclic two-phase framework.

## Conclusion

Extracellular phosphatase activity is considered to be the mechanism by which soil P_o_ is made available to plants and microorganisms through enzymatic catalysis. The production of extracellular phosphatases and their subsequent activity are governed by the P demand of the plant, as well as the availability of P_o_ substrate in the rhizosphere. Upon the plant's demand for P, non-equilibrium conditions develop between readily available dissolved P_i_ and the soil's labile P pool, resulting in an increase in P mineralization through root and microbial phosphatase activity. In addition to the catalytic mechanism, several factors determine phosphatase activity, such as the accessibility of P_o_ substrates, the pH-sensitive charge of phosphatases, and the adsorption of phosphatases and their substrates to colloidal and mineral soil surfaces. The catalysis of soil P_o_ is strongly influenced by physicochemical properties of soil, including the presence of organic and phosphate anions. It has been observed that the amount of dissolved P_i_ in soil exerts a bimodal effect on extracellular phosphatase activity (i) directly, when P_i_ anions are excessive, they act as competitive inhibitors of phosphatases, preventing them from binding to substrates, and (ii) indirectly, by reducing their adsorption on soil surfaces. In addition, the availability of water influences the interactions between phosphatases and soil organic matter and mineral surfaces, hence controlling the catalysis and adsorption processes. The lack of water availability leads to diffusion limitations, which, in turn, affect the hydrolysis of soil P_o_ by phosphatases, and thereby facilitate their adsorption to the soil matrix.

The exudation of metabolic products from plant roots (e.g., mucilage, LMWOAs) increases soil P availability through alteration of soil physicochemical conditions. The hydraulic and physicochemical effects of mucilage and LMWOAs influence the process of soil P acquisition by phosphatase activity, and the mobilization of insoluble P compounds. Based on our conceptual framework, root exudation serves as a controlling factor that regulates the acquisition and restoration of P within the soil matrix and is responsible for driving the two phases of P_o_-phosphatase interactions. This conceptual framework provides both theoretical- and process-based insights into the dynamics of soil P. Phosphatases are secreted by plant roots to meet their requirement for P, particularly when dissolved P_i_ is insufficient in the soil. The spatial and temporal shift between P acquisition and restoration affects the dynamics of P, implying a demand-driven strategy for resource acquisition by plants. As root exudation is largely controlled by plants, it is often poorly understood in its regulation, which makes it difficult to predict the transition from an acquiring phase I to a restoring phase II. To develop a detailed quantitative description and a reliable model of soil phosphatase activity, and thus P_o_ and P_i_ dynamics, future studies are needed that investigate the spatiotemporal heterogeneity of both phases of soil P cycling.

## Author Contributions

AM contributed to the conceptualization and drafting of this review manuscript. MD, SL, and EB provided critical feedback, improved and further developed a concept, suggested revisions, and approved the manuscript. All authors contributed to the article and approved the submitted version.

## Funding

AM acknowledges support from the Open Access Publication Funds of the Göttingen University. EB acknowledges support from the priority program 2089 Rhizosphere Spatiotemporal Organization–A Key to Rhizosphere Functions funded by the Deutsche Forschungsgemeinschaft (DFG, German Research Foundation) (Project No. 403664478). The Robert Bosch Foundation provided funding for MD and part of the publication fee *via* the Junior Professorship 2017 grant.

## Conflict of Interest

The authors declare that the research was conducted in the absence of any commercial or financial relationships that could be construed as a potential conflict of interest.

## Publisher's Note

All claims expressed in this article are solely those of the authors and do not necessarily represent those of their affiliated organizations, or those of the publisher, the editors and the reviewers. Any product that may be evaluated in this article, or claim that may be made by its manufacturer, is not guaranteed or endorsed by the publisher.
